# Impact of Chronotype and Mediterranean Diet on the Risk of Liver Fibrosis in Patients with Non-Alcoholic Fatty Liver Disease

**DOI:** 10.3390/nu15143257

**Published:** 2023-07-23

**Authors:** Gabriele Castelnuovo, Nuria Perez-Diaz-del-Campo, Chiara Rosso, Marta Guariglia, Angelo Armandi, Aurora Nicolosi, Gian Paolo Caviglia, Elisabetta Bugianesi

**Affiliations:** 1 Department of Medical Sciences, University of Turin, 10126 Turin, Italy; gabriele.castelnuovo@unito.it (G.C.); chiara.rosso@unito.it (C.R.); marta.guariglia@unito.it (M.G.); angelo.armandi@unito.it (A.A.); aurora.nicolosi@unito.it (A.N.); gianpaolo.caviglia@unito.it (G.P.C.); 2 Metabolic Liver Disease Research Program, I. Department of Medicine, University Medical Center of the Johannes Gutenberg-University, 55131 Mainz, Germany; 3 Gastroenterology Unit, Città della Salute e della Scienza—Molinette Hospital, 10126 Turin, Italy

**Keywords:** chronotype, non-alcoholic fatty liver disease, liver fibrosis, Mediterranean diet

## Abstract

Late chronotype, the individual’s aptitude to perform daily activities late in the day, has been associated with low adherence to the Mediterranean diet (MedDiet) and metabolic syndrome. The aim of this work was to investigate the potential association of chronotype and adherence to the MedDiet with the liver fibrosis risk in patients with non-alcoholic fatty liver disease (NAFLD). Liver stiffness was assessed in 126 patients by FibroScan^®^530. Significant (F ≥ 2) and advanced (F ≥ 3) hepatic fibrosis were defined according to liver stiffness values ≥7.1 kPa and ≥8.8 kPa, respectively. Chronotype (MSFsc) was defined by the Munich Chronotype Questionnaire, and adherence to the MedDiet was defined by the Mediterranean diet score (MDS). Overall, the median age was 55 (46–63) years, and 57.9% of participants were male. The principal comorbidities were type-2 diabetes mellitus (T2DM) (26.1%), arterial hypertension (53.1%), dyslipidaemia (63.4%), obstructive sleep apnoea (5.5%) and depression (5.5%). Most subjects (65.0%) had intermediate + late chronotype and showed higher mid-sleep on workdays (*p* < 0.001) and on work-free days (*p* < 0.001) compared to those with early chronotype. In the logistic regression model, intermediate + late chronotype (*p* = 0.024), MDS (*p* = 0.019) and T2DM (*p* = 0.004) were found to be significantly and independently associated with the risk of both F ≥ 2 And F ≥ 3. We observed that the intermediate + late chronotype and low adherence to the MedDiet were associated with both significant and advanced liver fibrosis in patients with NAFLD.

## 1. Introduction

Non-alcoholic fatty liver disease (NAFLD) is a rapidly growing epidemic worldwide [1,2]. The full spectrum of NAFLD ranges from simple steatosis [3] to non-alcoholic steatohepatitis (NASH) [4], characterised by inflammation, hepatocyte lesions and varying degrees of fibrosis [5,6], leading to cirrhosis and hepatocellular carcinoma (HCC) [7].

The underlying mechanisms for the development of NAFLD are complex and involve multiple factors, including nutrient intake, genetic and epigenetic factors, physical activity and the composition of the microbiota [8,9]. The primary approach for NAFLD treatment is focused on modifying lifestyle habits. This involves promoting the Mediterranean diet (MedDiet), which is rich in polyphenols, carotenoids, fibre and polyunsaturated fatty acids, while avoiding refined and high-sugar foods, together with regular physical activity, with the aim of achieving weight loss and effectively managing the cardiometabolic risk factors associated with the metabolic syndrome [10,11] However, macronutrients and food choices can be adapted to the patient’s needs, as long as the principles of a healthy diet based on the Mediterranean model are respected [11].

Interestingly, sleep disorders have been reported to be common in patients with NAFLD [12]. Sleep patterns may influence circadian rhythms, which are endogenous to our bodies and play an important role in various biological functions and human metabolism. The expression of the circadian clock system is referred to as the ‘chronotype’ and has recently been used in association with increased risk of metabolic diseases, such as obesity and its comorbidities/complications [13,14]. In this regard, the dysregulation of the circadian clock has been associated with hepatic fat deposition and its progression to NASH both in animals [15,16,17] and recently in humans [17,18]. Importantly, the circadian clock can also interact with nutrients, influencing metabolism, gut microbiota and hunger/satiety [19].

**Figure 1:**
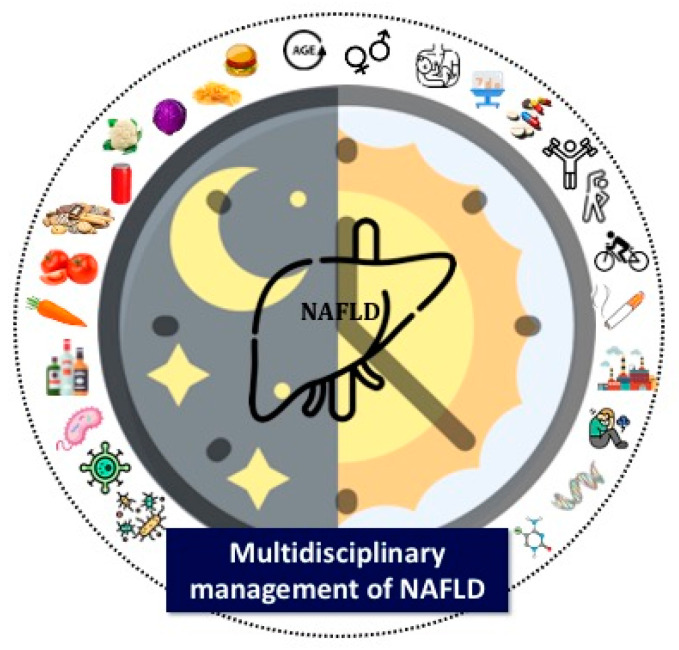
Multidisciplinary management of NAFLD. Environmental factors such as nutrient intake and exposure or physical activity, genetics, epigenetics or microbiota composition have been shown to be associated with NAFLD. Similarly, an individual’s chronotype influences the development of this disease, being important to consider it along with the aforementioned factors for an accurate and comprehensive management of these patients. Abbreviations: NAFLD, non-alcoholic fatty liver disease.

For these reasons, recent studies have focused on the importance of the chronotype, i.e., an individual’s aptitude to perform daily activities during the day, leading to the classification of individuals mainly into morning, intermediate and evening types [17]. Compared to early risers, evening types, who prefer to perform daily activities late in the day and going to bed and waking up later, are associated with poorer health status and are more likely to be overweight or obese [20], to be depressed and to have other risks of metabolic disease [21]. In this regard, individuals with an evening chronotype followed an unhealthy lifestyle, characterised by lower physical activity and adherence to the MedDiet, and had a higher body mass index (BMI) [22]. Moreover, in youth, adherence to a healthy diet is associated with morning chronotype and better overall sleep quality [23]. Hence, in recent years, chronotype has been recognised as an important tool for the modifications of the circadian rhythm and the study of the related metabolic diseases, especially obesity, which is strongly associated with the development of NAFLD. Therefore, in order to personalise treatment in patients with this disease, factors such as chronotype that impact or interact with other genetic or environmental features should be considered (Figure 1).

The purpose of this work was to investigate the impact of chronotype and adherence to the MedDiet on the risk of significant (F ≥ 2) and advanced (F ≥ 3) liver fibrosis in individuals with NAFLD.

## 2. Materials and Methods

### 2.1. Study Design and Participants

**Figure 2:**
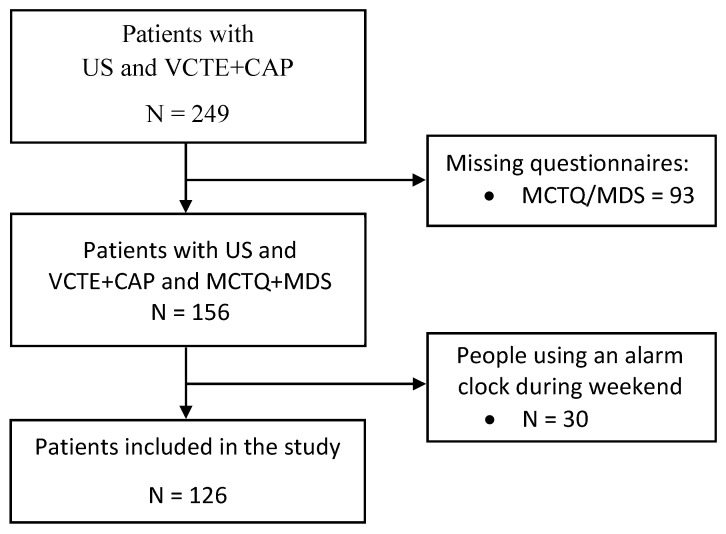
Recruitment flowchart. Abbreviations: MCTQ, Munich Chronotype Questionnaire; MDS, Mediterranean Diet Score; US, Ultrasound; VCTE + CAP, Vibration controlled transient elastography with assessment of Controlled attenuation parameter.

From a cohort of 249 consecutively per referral enrolled patients with a diagnosis of NAFLD already achieved by ultrasound (US), the present study included 126 individuals enrolled between October 2019 and October 2022 at the outpatients clinic of the Unit of Gastroenterology, Città della Salute e della Scienza di Torino—Molinette Hospital. This study was examined and approved by the Intercompany Ethics Committee A.O.U. Città della Salute e della Scienza di Torino—A.O. Ordine Mauriziano—A.S.L. Città di Torino. We included in the analysis 126 patients who underwent vibration controlled transient elastography (VCTE) with assessment of controlled attenuation parameter (CAP) (FibroScan^®^530, Echosens, Paris, France), who were administered the Munich Chronotype Questionnaire (MCTQ) and the Mediterranean Diet Score (MDS) (Figure 2). Subjects with other causes of liver disease such as chronic viral hepatitis (hepatitis B and C), autoimmune liver disease, hereditary haemochromatosis, α1-antitrypsin deficiency, Wilson’s disease or drug-induced liver damage and subjects with alcohol-induced liver disease, obtained with a self-reported negative history of alcohol abuse defined as a weekly ethanol consumption <140 g for women and <210 g for men [24], were excluded from the analysis. Subjects aged ≥ 18 years signed the informed consent for participation in the study.

### 2.2. Anthropometric, Biochemical and Comorbidity Assessment

After an overnight fast, blood pressure, weight, height and body mass index (BMI), calculated as the body weight divided by the squared height (kg/m^2^), were measured by qualified staff. Moreover, aspartate aminotransferase (AST), alanine aminotransferase (ALT) and gamma-glutamyl transferase (GGT), glucose, total cholesterol and triglyceride concentrations as well as comorbidities were collected from patient’s medical records. In particular, the diagnosis of diabetes and arterial hypertension was determined according to international guidelines [25,26]. On the other hand, dyslipidaemia is the failure to balance lipids such as cholesterol, low- and high-density lipoprotein and triglycerides. Subjects undergoing pharmacological treatment for lipid management were also considered dyslipidaemic [27,28].

### 2.3. Evaluation of Liver Status

VCTE and CAP were performed after overnight fasting to assess steatosis and possible liver stiffness (LS) on supine subjects with the right arm under the head and the right leg crossed with the left one. The examination probe (M or XL) was chosen according to the body condition between the 6th and 9th intercostal space. At least 10 snapshots of valid values were taken, of which the median was selected, and technically reliable measurements were considered on the basis of the interquartile range (IQR)/mean of less than 30%. LS was reported as kPa while CAP values were reported as db/m; the degree of liver fibrosis was classified into one of five groups (F0 to F4) according to established cut-offs [29]. In addition, another non-invasive test such as FIB-4 was calculated from each patient medical record according to the original formula [30]:

FIB-4: (Age (years) × AST (U/L))/(Platelets (109/L) × √ALT (U/L)) (1)

### 2.4. Chronotype Assessment

The Munich Chronotype Questionnaire (MCTQ), developed by Roenneberg et al. [31], quantifies the chronotype on the basis of the average sleep duration reported on workdays and work-free days. For chronotype calculation, the sleep duration on workdays (SDw) and on work-free days (SDf), the average weekly sleep duration (SDweek) and the mid-sleep on workdays (MSW) and on work-free days (MSF) were determined. MSF or MSW correspond to the start time of sleep plus half of its duration on work-free days and workdays, respectively.

MSF or MSW = sleep onset + sleep duration/2 (2)

The MCTQ calculates chronotype as MSF. However, since sleep requirements are not always met by generally depriving of minutes of sleep individuals who are used to setting the alarm clock during the week due to social compromises (e.g., work or study), when people sleep more on their work-free days (SDf > SDw), the chronotype is calculated by means of the MSF corrected for excessive sleep on work-free days due to sleep debt accumulated during the workdays (MSFsc).

MSFsc = MSF − (SDf − SDweek)/2

Consequently, if SDf > SDw, the chronotype was calculated as MSFsc. Otherwise, if SDF ≤ SDW, MSF was used as the chronotype marker. Therefore, to avoid possible bias due to poor compensation of sleep debt at the weekend, the MSFsc was calculated in people not using the alarm clock to wake up on work-free days [32]. The chronotype score represents circadian preferences, with higher values indicating an increasing tendency towards the evening type [33].

### 2.5. Mediterranean Diet Assessment

The MDS, first introduced by Trichopoulou et al. in 1995 [34], was used to assess the adherence to the MedDiet. The questionnaire is based on a 14-point screening, ranging from 0 to 14, where a higher final score indicates better adherence to the MedDiet [35].

### 2.6. Statistical Analysis

Continuous variables are expressed as means and standard deviation (SD) or as medians and interquartile ranges (IQR) depending on its distribution, while qualitative categorical variables were reported as absolute (*n*) and relative frequencies (%). Distribution of variables was assessed through the Shapiro–Wilk test, and outliers were also checked using boxplots. Groups were compared by Student’s *t*-tests for unpaired samples when data followed a normal distribution and the Mann–Whitney U test when data not show a normal distribution. Categorical variables were compared using the Chi-squared test.

Liver fibrosis was classified into 5 groups (from F0 to F4) according to established cut-off [29]. In the whole study population, liver fibrosis was classified into two different groups LS ≤ 7.0 vs. LS ≥ 7.1 according to established cut-off for significant fibrosis, while advanced liver fibrosis was classified into two different groups, LS ≤ 8.7 vs. LS ≥ 8.8. Consistent with the approach recommended for comparing chronotypes based on mid-sleep times derived from MCTQ within a sample, the MSFsc was divided into early, intermediate and late, by applying tertile splits [36]. Then, to carry out the analyses we evaluate the effect of tertile one versus tertile two + three.

To evaluate the potential association between both significant and advanced liver fibrosis risk (dependent variables) with intermediate + late chronotype (independent variable), logistic regression models were performed. Multivariable logistic regression analyses were adjusted for potential confounders, such as age, sex and body mass index; also, all the variables showing at least a marginal statistical trend (*p* < 0.10) at the univariate logistic analysis were also included in the model [37]. Data were expressed in Odds Ratio (OR) and confidence interval.

All *p*-values presented are two-tailed, and a *p* < 0.05 was considered statistically significant. Statistical analyses were carried out using the software STATA version 12.0 (Stata Corp College Station, TX, USA).

## 3. Results

General characteristics of participants are reported in Table 1. Overall, the median age of the cohort was 55 (46–63) years with 73 (57.93%) males and 53 (42.06%) females. Participants’ median BMI was 29.4 (range 26.2–32.5) kg/m^2^, and the principal comorbidities were type 2 diabetes mellitus (T2DM) (*n* = 33; 26.19%), arterial hypertension (*n* = 67; 53.17%), dyslipidaemia (*n* = 80; 63.49%), obstructive sleep apnoea syndrome (OSAS) (*n* = 7; 5.56%) and depression (*n* = 7; 5.56%). In addition, among patients with T2DM, three were treated with Sodium-glucose Cotransporter-2 Inhibitors, 2 with Thiazolidinedione and 1 with Glucagon-like peptide-1 receptor agonists.

As for the liver parameters, the median LS and CAP values were 5.1 kPa (range 4.4–6.0) and 302 dB/m (range 263–338), respectively, while FIB-4 was 0.98 (range 0.74–1.26). Moreover, 15.08% (*n* = 19) of the participants had F ≥ 2, while 10.32% (*n* = 13) had F ≥ 3. Patients’ biochemical parameters were also assessed. Interestingly, 221 min (range 195–267) was the median value of chronotype, and the median values of its variables were 450 min (range 410–490) for SDf, 405 min (range 360–450) for SDw and 420 min (range 372–456) for SDweek. In addition, the median MDS used to assed the adherence to MedDiet was 7 (range 5–8) (Table 1).

When analysing NAFLD patients according to chronotype, significant differences were observed only for MSF and MSW (*p* < 0.001 vs. *p* < 0.001, respectively); both parameters were significantly higher in patients with intermediate + late chronotype as compared to those with early chronotype. Conversely, no significant differences were observed for T2DM (*p* = 0.283), hypertension (*p* = 0.177), dyslipidaemia (*p* = 0.423), depression (*p* = 0.239) and OSAS (*p* = 0.650). Furthermore, neither LS (*p* = 0.149) nor CAP (*p* = 0.640) nor FIB-4 (*p* = 0.376) were statistically different between early and intermediate + late chronotype groups. Chronotype variables such as SDweek, SDw and SDf showed no significant differences between groups (*p* = 0.456, *p* = 0.695, *p* = 0.392, respectively). Lastly, the MDS showed no statistically significant differences between early and intermediate + late chronotype (*p* = 0.528) (Table 2).

Remarkably, for logistic regression analysis adjusted for MDS, sex, age, BMI, T2DM, OSAS, arterial hypertension and dyslipidaemia, only intermediate + late chronotype (OR = 9.29, 95% CI 1.33–64.50, *p* = 0.024), MDS (OR = 0.69, 95% CI 0.50–0.94, *p* = 0.019) and T2DM (OR = 7.6, 95% CI 1.92–30.05, *p* = 0.004) were as a result significantly and independently associated with F ≥ 2 (Table 3).

Similarly, in a logistic regression model adjusted for MDS, sex, age, T2DM and arterial hypertension, only intermediate + late chronotype (OR = 23.38, 95% CI 1.63–335.10, *p* = 0.020), MDS (OR = 0.63, 95% CI 0.43–0.93, *p* = 0.021) and T2DM (OR = 27.23, 95% CI 4.33–171.11, *p* < 0.001) were as a result significantly and independently associated with F ≥ 3 (Table 4).

## 4. Discussion

In this study, we demonstrated that both chronotype and MedDiet might impact on the risk of liver fibrosis. Specifically, we showed that the intermediate + late chronotype, together with poor adherence to the MedDiet, was independently and significantly associated with both F ≥ 2 and F ≥ 3.

The concept of MSFsc refers to an indicator of the preferred time of day for performing daily activities and may represent an important factor in the management of NAFLD and other metabolic disorders. Interestingly in our cohort, the intermediate + late chronotype showed higher MSW and MSF values, which serves as a sleep phase reference point. The higher values of these two variables means higher values of chronotype. The link between chronotype and liver has been previously reported in 87 patients with obesity, resulting in an evening chronotype associated with a more advanced NAFLD calculated with the Liver Fat Equation (*p* = 0.034), the Hepatic Steatosis Index (*p* < 0.001) and the Index of Non-Alcoholic Steatohepatitis (*p* = 0.014) [17]. Our results are therefore consistent with these results but also bring and reinforce for the first time the plausible importance of both chronotype and diet in the management of NAFLD [38].

NAFLD is a well-established risk factor for the onset of T2DM with a reciprocal interaction [39]. In a meta-analysis including 16 studies (*n* = 214,805) with 10,356 cases of incident T2DM, the risk of incident diabetes was increased two-fold in subjects with NAFLD (random-effects hazard ratio (HR) 2.22, 95% CI 1.84–2.60; *I*^2^ = 79.2%) [40]. Conversely, T2DM is an important risk factor for the progression of liver damage [41]. An evening chronotype may be also associated with increased risk for T2DM onset [21]: analyses performed on the 4589 subjects included in the DILGOM substudy of the 2007 National FINRISK Study showed an increased risk of T2DM (OR: 2.6, *p* < 0.001) and hypertension (OR: 1.3, *p* < 0.05) in individuals with an evening chronotype compared to those with morning chronotypes [42]. However, in our cohort, the relationship between intermediate + late chronotype and advanced liver fibrosis was mediated by T2DM (*p* = 0.283). Nevertheless, a bidirectional influence may be hypothesised, as T2DM is frequently accompanied by others symptoms such as obesity, neuropathic pain and nocturnal hypoglycaemia, among others, which may affect sleep quality [43,44].

Additionally, patients with NAFLD have increased risk for sleep disorders independently of other pathologies [45]. In this regard, sleep quality may be altered by hypertension, depression [46] and OSAS [47]. Data from a meta-analysis reported that patients with hypertension (OR: 1.48; *p* = 0.01) showed significantly poorer subjective sleep quality when analysing the Pittsburgh Sleep Quality Index results [48].

While the late chronotype is mainly studied in relation to obesity, metabolic syndrome, sarcopenia [49] and cardiovascular disease [50], early chronotype has been associated with a healthy lifestyle and, in particular, with lower consumption of sweets or sweeteners and ultra-processed fats (*p* = 0.030 and *p* = 0.035, respectively) [51]. The current management of patients with NAFLD is focused on lifestyle habits and dietary pattern improvement, as well as the control of the environmental factors [52]. However, in our cohort, no significant differences in adherence to MedDiet as well as in BMI and co-morbidities (hypertension, dyslipidaemia, diabetes and OSAS) have been found among groups.

To our knowledge, this is the first study to investigate the relationship between chronotype, diet and risk of liver fibrosis using the MCTQ questionnaires in patients with NAFLD. One of the main strengths of this research is that all patients included in the study were assessed by VCTE, Fibroscan in NAFLD patients, which is one of the most widely used and best validated tests [53]. Furthermore, these results can be considered together with age, gender, physical activity, genetics and other environmental factors for personalised and precision treatment of these patients [54,55].

On the other hand, one possible limitation of the present study is that the MCTQ takes into accounts only the time of sleep onset and awakening. However, many people have problems with sleep continuity, waking up and falling asleep several times during the night. Moreover, the MCTQ is not applicable in patients who use an alarm clock on their days off to wake up, just as it is not validated in shift workers [56]. Furthermore, the failure to use actimeters based the sleep calculation solely on the MCTQ, which does not take into account inertia, sleep latency, efficiency and actual sleep duration. Furthermore, the cross-sectional design of the study did not allow causal inferences to be made, so further studies on a larger number of individuals and different cohorts are needed. Another limitation is due to the sample division into chronotype categories using tertile splits, even if it is recommended to compare different chronotypes within a sample [36]. Thus, results may be susceptible to some biases; our results must be considered preliminary and require further verification.

## 5. Conclusions

In conclusion, this study shows that intermediate + late chronotype and low adherence to MedDiet were associated with significant and advanced liver fibrosis in patients with NAFLD. Therefore, it might provide the rational basis for a personalised management of patients with NAFLD taking into account not only patients’ diet and physical activity but also sleep characteristics that may represent an increased risk for the onset and progression of liver disease.

## Figures and Tables

**Table 1:** General characteristics of participants.

*n*	126
Sex (Male/Female)	73/53
Age (years)	55 (46; 63)
**Anthropometric characteristics**	
Body Mass Index (kg/m^2^)	29.4 (26.2; 32.5)
Diabetes *n* (%)	33 (26.19%)
Arterial hypertension *n* (%)	67 (53.17%)
Dyslipidaemia *n* (%)	80 (63.49%)
Depression *n* (%)	7 (5.56%)
Obstructive sleep apnoea *n* (%)	7 (5.56%)
**Liver and biomarkers assessment**	
Liver stiffness (kPa)	5.1 (4.4; 6)
Liver stiffness *n* (%)	
≤7 kPa (F0–F1)	107 (84.92%)
7.1–8.7 kPa (F2)	6 (4.76%)
8.8–10.3 Kpa (F3)	5 (3.97%)
≥10.4 (F4)	8 (6.35%)
CAP (dB/m)	302 (263; 338)
FIB-4	0.98 (0.74; 1.26)
AST (IU/L)	28 (24; 38)
ALT (IU/L)	39 (27; 56)
GGT (IU/L)	40 (26; 75)
Glucose (mg/dL)	98 (87; 112)
Triglycerides (mg/dL)	128.5 (100; 171)
Cholesterol (mg/dL)	200 (177; 228)
MSF (min)	240 (210; 285)
MSW (min)	201.5 (170; 235)
SDf (min)	450 (410; 490)
SDw (min)	405 (360; 450)
SDweek (min)	420 (372; 456)
Chronotype (min)	221 (195; 267)
Mediterranean diet score	7 (5; 8)

Data are shown as *n* (percent) or median (IQR). Abbreviations: ALT, Alanine Aminotransferase; AST, Aspartate Aminotransferase; CAP, Controlled Attenuation Parameter; FIB-4: Fibrosis-4 score; GGT, Gamma-Glutamyl Transferase; MSF, Mid-Sleep on work-free days; MSW, Mid-Sleep on workdays; SDf, Sleep duration on work-free days; SDw, Sleep duration on workdays; SDweek, Average weekly sleep duration.

**Table 2:** Characteristics of participants according to the chronotype.

	Early	Intermediate + Late	*p*-Value
*n*	44	82	
Sex *n* (%)			
Male	23 (52.27%)	50 (60.98%)	0.345
Female	21 (47.73%)	32 (39.02%)	
Age (y)	57 (49; 62)	51.5 (42; 63)	0.301
Diabetes *n* (%)			
No	35 (79.55%)	58 (70.73%)	0.283
Yes	9 (20.45%)	24 (29.27%)	
Hypertension *n* (%)			
No	17 (38.64%)	42 (51.22%)	0.177
Yes	27 (61.36%)	40 (48.78%)	
Dyslipidaemia *n* (%)			
No	14 (31.82%)	32 (39.02%)	0.423
Yes	30 (68.18%)	50 (60.98%)	
Depression *n* (%)			
No	43 (97.73%)	76 (92.68%)	0.239
Yes	1 (2.27%)	6 (7.32%)	
Obstructive sleep apnoea *n* (%)			
No	41 (93.18%)	78 (95.12%)	0.650
Yes	3 (6.82%)	4 (4.88%)	
Body Mass Index (kg/m^2^)	29.05 (26.8; 32.4)	29.4 (25.7; 32.8)	0.619
Stiffness *n* (%)			
≤7 kPa (F0–F1)	41 (93.18%)	66 (80.49%)	0.149
7.1–8.7 kPa (F2)	2 (4.55%)	4 (4.88%)	
8.8–10.3 kPa (F3)	1 (2.27%)	4 (4.88%)	
≥10.4 kPa (F4)	0 (0%)	8 (9.76%)	
CAP (dB/m)	305.5 (267.5; 337.5)	300 (260; 340)	0.640
FIB-4	1.01 (0.84; 1.24)	0.94 (0.67; 1.30)	0.376
AST (IU/L)	30.5 (23; 40.5)	27.5 (24; 37)	0.498
ALT (IU/L)	38.5 (29; 59)	39 (27; 51)	0.506
GGT (IU/L)	40.5 (27; 68)	39 (26; 76)	0.840
Glucose (mg/dL)	101.5 (87; 111.5)	95 (86; 117)	0.595
Triglycerides (mg/dL)	130 (91; 173)	128 (107; 171)	0.764
Cholesterol (mg/dL)	205.94 (49.99)	203.65 (40.92)	0.804
SDweek (min)	429 (372.5; 477.5)	416.5 (372; 450)	0.456
MSF (min)	195 (180; 225)	272.5 (235; 310)	<0.001
MSW (min)	170 (120; 190)	225 (200; 255)	<0.001
SDw (min)	410 (360; 450)	400 (360; 440)	0.695
SDf (min)	458.34 (87.66)	445.65 (73.99)	0.392
Mediterranean diet score	6.84 (2.07)	7.09 (2.22)	0.528

Data are shown as *n* (percent), mean (SD) or median (IQR). Abbreviations: ALT, Alanine Aminotransferase; AST, Aspartate Aminotransferase; CAP, Controlled Attenuation Parameter; FIB-4: Fibrosis-4 score; GGT, Gamma-Glutamyl Transferase; MSF, Mid-Sleep on work-free days; MSW, Mid-Sleep on workdays; SDf, Sleep duration on work-free days; SDw, Sleep duration on workdays; SDweek, Average weekly sleep duration.

**Table 3:** Univariate and multivariate analysis for risk of significant liver fibrosis (F ≥ 2) according to chronotype.

	OR (95% CI)	*p*-Value	OR (95% CI)	*p*-Value	Pseudo R-Squared	*p*-Value Model
Model 1					40.39	<0.001
Intermediate + late chronotype	3.31 (0.90; 12.07)	0.069	9.29 (1.33; 64.50)	0.024		
Mediterranean diet score	0.74 (0.57; 0.94)	0.018	0.69 (0.50; 0.94)	0.019		
Female	0.77 (0.28; 2.11)	0.617	0.33 (0.71; 1.57)	0.167		
Age	1.04 (0.99; 1.09)	0.059	1.00 (0.94; 1.07)	0.795		
Body Mass Index	1.07 (0.99; 1.16)	0.063	1.02 (0.89; 1.16)	0.751		
Diabetes	9.4 (3.19; 27.82)	<0.001	7.60 (1.92; 30.05)	0.004		
Obstructive sleep apnoea	9.24 (1.88; 45.41)	0.006	5.55 (0.43; 70.91)	0.187		
Hypertension	5.85 (1.61; 21.27)	0.007	5.16 (0.73; 36.51)	0.100		
Dyslipidaemia	3.58 (0.98; 13.04)	0.053	4.00 (0.65; 24.35)	0.132		
Depress	2.4 (0.43; 13.38)	0.318	-	-		

**Table 4:** Univariate and multivariate analysis for risk of advanced liver fibrosis (F ≥ 3) according to chronotype.

	OR (95% CI)	*p*-Value	OR (95% CI)	*p*-Value	Pseudo R-Squared	*p*-Value Model
Model 1					45.77	<0.001
Intermediate + late chronotype	7.37 (0.92; 58.71)	0.059	23.38 (1.63; 335.10)	0.020		
Mediterranean diet score	0.79 (0.59; 1.05)	0.107	0.63 (0.43; 0.93)	0.021		
Female	0.58 (0.16; 1.99)	0.388	0.23 (0.03; 1.51)	0.126		
Age	1.05 (0.99; 1.11)	0.089	1.00 (0.94; 1.07)	0.809		
Body Mass Index	1.04 (0.95; 1.14)	0.381	-	-		
Diabetes	22.75 (4.70; 110.11)	<0.001	27.23 (4.33; 171.11)	<0.001		
Obstructive sleep apnoea	3.92 (0.68; 22.67)	0.126	-	-		
Hypertension	5.59 (1.18; 26.40)	0.030	5.68 (0.54; 59.52)	0.147		
Dyslipidaemia	2.04 (0.53; 7.85)	0.296	-	-		
Depress	1.48 (0.16; 13.40)	0.724	-	-		

## Data Availability

Data available on request due to privacy/ethical restrictions.
